# Food acquisition methods and correlates of food insecurity in adults on probation in Rhode Island

**DOI:** 10.1371/journal.pone.0198598

**Published:** 2018-06-08

**Authors:** Kimberly R. Dong, Alice M. Tang, Thomas J. Stopka, Curt G. Beckwith, Aviva Must

**Affiliations:** 1 Department of Public Health and Community Medicine, Tufts University School of Medicine, Boston, MA, United States of America; 2 The Miriam Hospital and the Division of Infectious Diseases, Warren Alpert Medical School, Brown University, Providence, RI, United States of America; TNO, NETHERLANDS

## Abstract

**Background:**

Individuals under community corrections supervision may be at increased risk for food insecurity because they face challenges similar to other marginalized populations, such as people experiencing housing instability or substance users. The prevalence of food insecurity and its correlates have not been studied in the community corrections population.

**Methods:**

We conducted a cross-sectional study in 2016, surveying 304 probationers in Rhode Island to estimate the prevalence of food insecurity, identify food acquisition methods, and determine characteristics of groups most at-risk for food insecurity. We used chi-square and Fisher’s exact tests to assess differences in sociodemographics and eating and food acquisition patterns, GIS to examine geospatial differences, and ordinal logistic regression to identify independent correlates across the four levels of food security.

**Results:**

Nearly three-quarters (70.4%) of the participants experienced food insecurity, with almost half (48.0%) having very low food security. This is substantially higher than the general population within the state of Rhode Island, which reported a prevalence of 12.8% food insecurity with 6.1% very low food security in 2016. Participants with very low food security most often acquired lunch foods from convenience stores (and less likely from grocery stores) compared to the other three levels of food security. Participants did not differ significantly with regards to places for food acquisition related to breakfast or dinner meals based upon food security status. In adjusted models, being homeless (AOR 2.34, 95% CI: 1.31, 4.18) and depressed (AOR 3.12, 95% CI: 1.98, 4.91) were independently associated with a greater odds of being in a food insecure group. Compared to having help with meals none of the time, participants who reported having meal help all of the time (AOR 0.28, 95% CI: 0.12, 0.64), most of the time (AOR 0.31, 95% CI: 0.15, 0.61), and some of the time (AOR 0.54, 95% CI: 0.29, 0.98) had a lower odds of being in a food insecure group. Food insecure participants resided in different neighborhoods than food secure participants. The highest density of food insecure participants resided in census tracts with the lowest median incomes for the general population. The areas of highest density for each level of food security for our participants were in the census tracts with the lowest levels of full-time employment for the general population.

**Conclusions:**

The prevalence of food insecurity and very low food security were markedly higher in our probation population compared to the general RI population. These findings suggest that access to food on a regular basis is a challenge for adults on probation. Depression and being homeless were independently associated with a greater odds of being in a food insecure group. In addition to intervening directly on food insecurity, developing interventions and policies that address the contributing factors of food insecurity, such as safe housing and treatment for depression, are critical.

## Introduction

Food insecurity, which is defined as uncertain or limited availability of nutritionally adequate or safe food or the inability to acquire personally acceptable foods in socially acceptable ways,[1] can result in poorer quality of dietary intake, which can lead to further health decrements. Very low food security is defined as a household with at least one individual that has reduced food intake and eating patterns that were disrupted over the year due to lack of money or other resources for food.[1] In 2016, 12.3% of US households (or about 15.6 million households) were food insecure at some point in the year and of that, 4.9% (or about 6.1 million households) had very low food security.[1]

**Fig 1:**
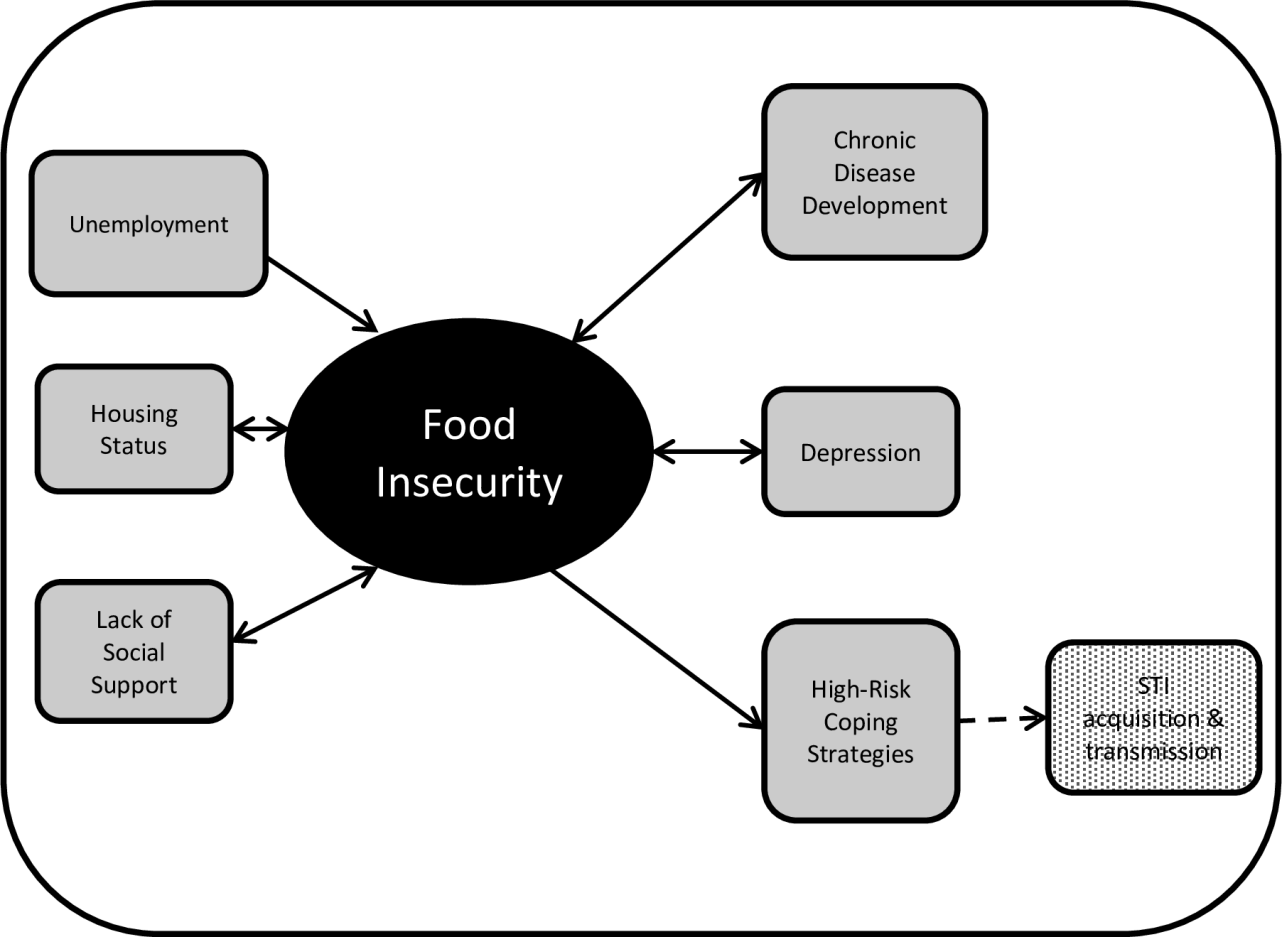
Conceptual framework for the relationships between potential correlates and outcomes of food insecurity. Many of these relationships can be bidirectional with food insecurity, which adds to the complexity of this framework.

Individuals under community corrections supervision, specifically probation or parole, face challenges with unemployment and underemployment due to their criminal records.[2, 3] These economic constraints may make accessing adequate food, safe housing, and healthcare challenging and potentially contribute to health disparities. One in 66 adults (about 1.5% of the population) was on probation in the US at the end of 2015[4] but little is known about the food security and health status of this group. The limited literature on probationers shows that they experience poor health outcomes, such as mental health issues,[5] and many engage in HIV-risk behaviors[6, 7] and substance use.[8–10] Individuals under probation supervision may be at increased risk for food insecurity because of financial hardships and may intersect with other marginalized populations, such as the homeless or substance using populations. Studies in these groups have found associations between food insecurity and chronic health conditions, such as obesity[11–14] and hypertension,[15–17] depression[18, 19] or other mental health conditions,[20] and behaviors that increase the risk for acquisition and transmission of infectious diseases, like HIV and hepatitis C (Fig 1).[21–23]

The prevalence of food insecurity and its correlates have not been studied in the community corrections population. Research that examines the prevalence and risk factors of food insecurity in this population is important in order to determine the magnitude of the problem, identify the people most at risk, and develop more targeted interventions and programs. The primary objectives of this study were to estimate the prevalence of food insecurity, determine primary mechanisms for food acquisition, and identify independent correlates of food insecurity in adults on probation. We hypothesized that the prevalence of food insecurity in adults on probation would be greater than in the general population.

## Materials and methods

### Study design and participants

Rhode Island has the second highest rate of community corrections supervision in the nation[24] with 23,823 adults on probation (or about 2.8% of the state population).[25] There are 11 probation offices across the state. Our study took place in one probation office in Rhode Island which supervises 12% of the people under community corrections in the state. We enrolled English speaking adults (≥18 years) that were under probation supervision at this office between July and October 2016 for this cross-sectional study. Because individuals on parole comprised only three percent of the individuals with regular visits at this office, we limited our study population to individuals on probation. We recruited a convenience sample of adults on probation two days per week during the study timeframe. Participants were recruited by study personnel in the waiting room of the probation office or by referral from probation officers. At the time of our study, there were approximately 800 individuals under active supervision at this probation office.

We administered an Audio Computer-Assisted Self-Interview (ACASI) survey, a method which has been shown to elicit more accurate responses to questions about sensitive risk behaviors compared to face-to-face interviews.[26–28] Participants self-administered the questionnaires on individual laptops with each participant listening to the survey questions and responses through their own headphones. The interview was designed to take approximately 30 minutes and began with a brief tutorial for participants to understand how to navigate the ACASI questionnaire. The ACASI system provided direct data entry of the participants’ survey responses. For each question, participants responded by pressing a number on the keyboard or using a mouse to click on the response. Each question also had the options to refuse or respond “Don’t Know”. To ensure confidentiality of survey responses, all ACASI surveys were administered in a private room at the probation office without probation officers present. Each participant received a $25 gift card as compensation for their time. The study was approved by two Institutional Review Boards, The Miriam Hospital and the Rhode Island Department of Corrections. Written informed consent was obtained from each participant.

### Food security

The food security status of participants, the primary outcome of interest, was measured using the USDA 10-Item US Adult Food Security Module.[29] The food security survey asks ten indicator questions that address three domains: 1) the respondents’ perception of uncertain or inadequate food access, 2) compromised eating patterns (such as decreasing portion sizes or meal skipping), and 3) physical consequences (such as hunger and weight loss) that might result from difficulty with meeting basic food needs due to lack of money or resources. The response timeframe was asked “within the last 30 days” in case participants recently initiated probation supervision and to measure current food security status. A point is given for each affirmative response and all scores were combined to create a composite variable with possible total scores ranging from 0 to 10. The food insecurity score was analyzed as a categorical variable defined as high food security (score 0), marginal food security (score 1 to 2), low food security (score 3 to 5), and very low food security (score 6 to 10), based upon USDA guidelines.[29]

### Food acquisition, meal patterns, and meal support

Methods for food acquisition and meal patterns were also assessed. For food acquisition, participants were asked, “*Where do you usually get food for [meal]*”, where this was asked separately for breakfast, lunch, and dinner. Response choices for breakfast, lunch, and dinner were obtained based on formative research with this population,[30] which included: “convenience store”, “fast food restaurant, like McDonald’s, Burger King, Popeyes, Taco Bell, Subway”, “local neighborhood market, like Shop A, Shop B, Shop C”, “food bank or another place where food is provided for free”, “large grocery store, like Shop D, Shop E, and Shop F”, “mobile food truck”, “farmer’s market”, or “I don’t eat [meal]”. The names of most food establishments have been redacted and replaced with “Shop” to maintain the confidentiality of our participants. Participants were also asked about whether they currently participated in the Supplemental Nutrition Assistance Program (SNAP, formerly known as food stamps) and if they ate a meal from a community program in the last 30 days as proxies for public social support for food. Participants were also asked to assess the healthfulness of their diet by responding to the question, “*In general*, *in your opinion*, *how healthy is your overall diet*?*”* with response options of “excellent”, “very good”, “good”, “fair”, or “poor”.

We were interested in the role of social support on food insecurity. We used meal support and meal preparation as proxies for private social support. We hypothesized that preparing one’s own meals would be a marker for lack of social support since a study among elderly men found that higher levels of social support were associated with a higher likelihood of not usually cooking one’s own meals.[31] The frequency of meal support was assessed by the question, “*How often is someone available to prepare your meals if you were unable to do this yourself*?*”* with response options of “none of the time”, “a little of the time”, “some of the time”, “most of the time”, or “all of the time”. Meal preparation was assessed by asking, *“Are you responsible for preparing most of your meals*?*”* with response options of “yes” or “no”.

### Other correlates

We collected information on known or potential risk factors for food insecurity. The ACASI questionnaire included questions on sociodemographics, correctional supervision history, depression, hazardous alcohol use, and illicit drug use. Sociodemographic items included gender, age, race, ethnicity, education, marital status, access to a car, housing status, residential address, employment status, annual income, number of children, and number of children currently residing with the participant. Correctional supervision history was queried with, *“What is the total length of time that you have ever been under correctional supervision”* and *“How long have you currently been on probation*?*”* Depressive symptoms were assessed using the Center for Epidemiologic Studies Depression Scale (CES-D) with a cutoff score of ≥16 to define depression.[32] Hazardous alcohol use was assessed using the Alcohol Use Disorders Identification Test (AUDIT), with a score of ≥8 as the cutoff for hazardous alcohol use.[33] Illicit drug use was assessed by determining current drug use (in the past 30 days), types of drugs ever and currently used, frequency of current use, and ever injected drugs (yes/no).

### Residential neighborhood

Participants were asked to provide the address at which they resided most often in the last 30 days. If participants were not willing to provide their address, they were asked for the intersecting cross-streets nearest to their residential address. The responses to the address and intersections questions were provided as field text in the ACASI for participants to type their responses.

### Analyses

Differences in sociodemographic characteristics, alcohol use, and drug use by the four levels of food security status were determined using chi-square or Fisher’s Exact tests for categorical variables and the Kruskal Wallis test for age (continuous variable). To determine the significant correlates of food insecurity, unadjusted proportional odds ordinal logistic regression models were constructed between each covariate and the four levels of the dependent variable, food security. All covariates that were statistically significant or approached statistical significance at p<0.10 in the unadjusted analysis were considered for the final adjusted ordinal logistic regression model. The covariates included in the final adjusted model included being homeless, depressed, and meal support. We used log likelihood ratio tests to compare nested to full models to determine the final adjusted ordinal logistic regression model. We also conducted likelihood ratio tests and the Brant tests to ensure we did not violate the proportional odds assumption. Odds ratios from ordinal logistic regression models can be interpreted as the odds of being in higher levels of the outcome (food security) given a particular exposure compared to the odds of being at lower levels of the outcome in the absence of that particular exposure.[34, 35] To determine the effect of gender and race/ethnicity on the correlates of food insecurity, we tested for interactions by generating a cross-product term in the regression models. Based on the results of the analyses, gender and race/ethnicity were not effect modifiers in the relationships between identified covariates and food security and were thus dropped from the models. Stata v13 (StataCorp, College Station, TX) was used for all statistical analyses.

We used a geographic information system (GIS) to examine the geospatial distribution of food security status using Kernel density estimates. We first geocoded the place of residence for all study participants to obtain latitude and longitude measures that facilitated mapping of participants by food security status. Next, we calculated Kernel density estimates to create a smooth, interpolated surface (i.e., a “heat map”) for each level of food security. This also served to geo-mask the precise location of study participants’ place of residence, which preserved confidentiality. Heat maps presented the density of participants per square mile for each of the four categories of food security status. We also mapped the median income and employment status of the general population that resided in the census tracts within our study frame. The median income and employment information data came from the American Fact Finder 2016 American Community Survey.[36] The employment data is represented as adults aged 16 to 64 years who were employed full time for one year and were normalized by the total population aged 16 to 64 years. We used ArcGIS v10.5 (Environmental Systems Research Institute, Redlands, CA) to conduct spatial analyses.

## Results

A total of 304 participants were enrolled in this study. Approximately 20% refused to participate at the time of recruitment because the timing was inconvenient with transportation home or other appointments. A smaller percentage (~5%) were not enrolled because they were non-English speaking. Probation officers reported that the individuals participating in this study were, in their estimation, generally representative of the individuals reporting to this office based upon gender, race, and socioeconomic status and did not differ from those who were unable or refused to participate.

Overall characteristics of the 304 participants in this study are summarized in Table 1, column 1. The median age was 36 years (interquartile range 27–47), 53% were non-Hispanic white, 28% were female, and 47% were never married. The highest level of education attained for most participants was high school (42%). Only one-quarter of the participants had a child currently living with them, 22% had a full-time job, almost two-thirds (63%) had an individual annual income of ≤ $10,000, and 28% always had access to a car. Household size was not different based upon food insecurity status (not reported in Table 1). Almost three-quarters (73%) of participants received SNAP benefits. A majority of participants currently smoked tobacco everyday (72%) and 39% reported using marijuana in the last 30 days. Current drug use in the past 30 days was reported by 19% of our participants and 24% currently consumed alcohol at a hazardous level. Depression was identified in 56% of participants. The prevalence of food insecurity in this study was 70%; almost half (48%) had very low food security. The prevalence of food insecurity and very low food security reported in the past 30 days in this study are both substantially higher compared to the state prevalence (12.8% and 6.1%, respectively), reported in the prior 12 months.[1]

**Table 1:** Sociodemographic characteristics: Adults on probation in Rhode Island by food security status, 2016 (n = 304). N (%) or median (IQR)^a^.

	All (n = 304)	High Food Security (n = 51)	Marginal Food Security (n = 39)	Low Food Security (n = 68)	Very Low Food Security (n = 146)
Age, years (19–65 years)	36 (27, 47)	36 (27, 47)	34 (24, 53)	34 (26, 49)	36 (27, 46)
Male	220 (72)	38 (75)	33 (85)	45 (66)	104 (71)
Race/ethnicity^b^					
Black, non-Hispanic	45 (15)	7 (14)	8 (21)	9 (13)	21 (14)
Hispanic or Latino, any race	64 (21)	10 (20)	4 (10)	17 (25)	33 (23)
White, non-Hispanic	161 (53)	33 (65)	21 (54)	34 (51)	73 (50)
Other	33 (11)	1 (2)	6 (15)	7 (10)	19 (13)
Homeless**	70 (23)	4 (8)	5 (13)	13 (19)	48 (33)
Current Time on Probation					
<1 year	114 (38)	20 (39)	14 (36)	21 (31)	59 (40)
1 year to <18 months	39 (13)	8 (16)	6 (15)	9 (13)	16 (11)
18 months to <2 years	25 (8)	5 (10)	2 (5)	4 (6)	14 (10)
2 years or more	126 (41)	18 (35)	17 (44)	34 (50)	57 (39)
Education					
Less than High School/ no GED	101 (33)	19 (37)	12 (31)	22 (33)	48 (33)
High School/GED	129 (42)	17 (33)	18 (46)	29 (43)	65 (45)
Trade/technical school	19 (6)	5 (10)	1 (3)	6 (9)	7 (5)
Some college/college degree/graduate school	54 (18)	10 (20)	8 (20)	10 (15)	26 (17)
Relationship Status					
Never Married	143 (47)	22 (43)	21 (54)	35 (51)	65 (45)
Married	22 (7)	4 (8)	2 (5)	3 (4)	13 (9)
Divorced/Widowed	53 (17)	12 (24)	4 (10)	13 (19)	24 (16)
Separated	30 (10)	3 (6)	5 (13)	2 (3)	20 (14)
Living with Partner	56 (18)	10 (20)	17 (18)	15 (22)	24 (16)
Children living with Participant (n = 212 have children)	75 (25)	16 (31)	7 (18)	21 (31)	31 (21)
Sources of Income (all that apply)					
Full-time Job**	66 (22)	18 (35)	13 (33)	14 (21)	21 (14)
Part-time Job	58 (19)	8 (16)	3 (8)	19 (28)	28 (19)
SSI, Disability, VA	89 (29)	15 (29)	12 (31)	18 (26)	44 (30)
Individual Annual Income					
≤ $5000	133 (44)	21 (41)	14 (36)	30 (44)	68 (47)
$5001 to $10,000	59 (19)	8 (16)	8 (21)	14 (21)	29 (20)
$10,001 to $20,000	38 (13)	5 (10)	7 (18)	9 (13)	17 (12)
> $20,000	53 (17)	14 (27)	6 (15)	12 (18)	21 (14)
Don’t Know/Refused	21 (7)	3 (6)	4 (10)	3 (4)	11 (7)
Access to Car					
Always**	85 (28)	25 (49)	13 (33)	21 (31)	26 (18)
Sometimes	102 (34)	12 (24)	14 (36)	23 (34)	53 (36)
Never	117 (39)	14 (27)	12 (31)	24 (35)	67 (46)
SNAP Participation	223 (73)	33 (65)	26 (67)	52 (76)	112 (77)
Current Smoker					
Everyday	142 (72)	21 (68)	11 (61)	29 (74)	81 (74)
Some days	39 (20)	6 (19)	4 (22)	8 (21)	21 (19)
Not at all	17 (9)	4 (13)	3 (17)	2 (5)	8 (7)
Hazardous alcohol level	74 (24)	10 (20)	7 (18)	19 (28)	38 (26)
Current marijuana use (last 30 days)	119 (39)	18 (35)	12 (31)	26 (38)	63 (43)
Current drug use (last 30 days)	58 (19)	11 (22)	5 (13)	14 (21)	28 (19)
Ever drug use	189 (62)	30 (59)	18 (46)	41 (60)	100 (68)
IDU ever*	54 (18)	9 (18)	1 (3)	12 (18)	32 (22)
Depressed***	169 (56)	12 (24)	20 (51)	31 (46)	106 (73)

^a^IQR = Interquartile Range: 25^th^ percentile and 75^th^ percentile

^b^Other for race/ethnicity includes multiracial, American Indian or Alaskan Native, Native Hawaiian or Pacific Islander, and “other”. If participants selected “other” for the response option to race, we did not have a follow-up question to determine the race. No participants reported Asian for race and one participant refused to answer the race question.

P-values determined by the chi-square test for categorical variables. P-values show the homogeneity of proportions across categories of food security

*p-value <0.05

**p-value<0.01

***p-value<0.001

GED = General Education Development (High school equivalency diploma); SNAP = Supplemental Nutrition Assistance Program; IDU = Injection Drug Use

When comparing participant characteristics by the four levels of food security (Table 1), we found that participants who had very low food security were more likely to be homeless (33%) than the other levels of food security (19% for low food security, 13% for marginal food security, and 8% for high food security, p<0.01). There were significant differences in participants having access to a car between the four levels of food security, with those in the highest level of food security most likely to always have access (49%). High food security participants were more often employed with a full-time job (35%) compared to the other levels of food security (p<0.01). Depression was more common in participants with very low food security (73%) compared to the other levels of food security (46% for low food security, 51% for marginal food security, and 24% for high food security, p<0.001).

**Fig 2:**
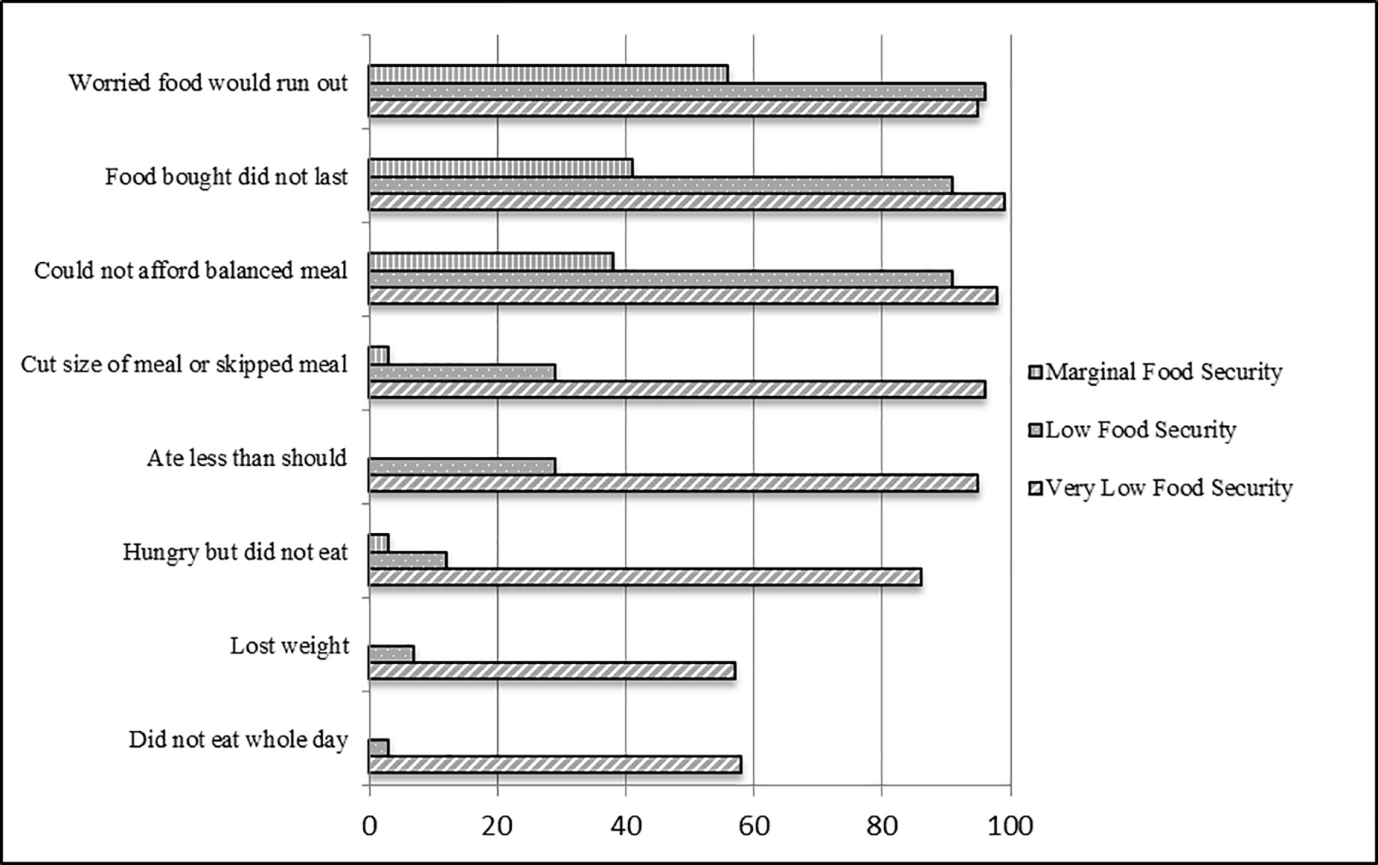
Percentage of adults on probation reporting each indicator of food insecurity in the last 30 days, by food security status (n = 304), Rhode Island, 2016. For each of the ten indicators for food insecurity, the percentages of each level of food security that reported an affirmative response to each indicator are shown. For the indicators, cut the size of a meal or skipped a meal and did not eat for a whole day, the mean number of days that individuals that provided an affirmative response to these indicators are represented by food security level.

The percentage of participants reporting the presence of each food insecurity indicator in the last month are depicted in Fig 2 by food security status. More than half (58%) of participants with very low food security and 3% of those with low food security reported not eating for a whole day. For both groups combined, participants reported not eating for a whole day in the last month, on average for nine days. More than half (57%) of participants with very low food security and 7% of those with low food security reported losing weight in the last 30 days from insufficient food intake. The majority (86%) of very low, 12% of low, and 3% of marginal food secure participants reported hunger and not being able to eat at some point the last 30 days. More than half of the total participants reported eating less than they should (52%) and cutting the size of or skipping a meal (53%). The average number of days in the last month this occurred was 11 days for participants with very low food security, five days for participants with low food security, and one day for participants with marginal food security.

Self-reported diet quality and methods of food acquisition by level of food security are shown in Table 2. There were significant differences in self-reported dietary quality by level of food security (p<0.001), and 27% of those with very low food security rated their dietary quality as poor compared to 7% in low and 8% for both marginal and high food security. There was a significant association between food insecurity status and eating meals at a community program in the last month, with 23% reporting this in the very low food security group and 6–10% in the high, marginal, and low food security groups (p<0.001). There were also significant differences in places to most likely acquire lunch foods based upon level of food security (p<0.05), with participants with very low food security more likely to acquire lunch foods from convenience stores and less likely from grocery stores compared to the other three levels of food security. Availability to have help with meals significantly differed based upon food security status (p<0.001) with participants with very low food security most likely to have help “none of the time” compared to the other three levels of food security. Participants’ responsibility for preparing their own meals differed significantly by food security status (p<0.001) with very low and low food security participants more likely to be responsible for preparing their own meals (92% and 91% respectively) compared to marginal and high food security (69% and 82% respectively).

**Table 2:** Self-reported dietary quality and food acquisition methods by food security status: Adults on probation in Rhode Island, 2016, N (%).

	All (n = 304)	High Food Security (n = 51)	Marginal Food Security (n = 39)	Low Food Security (n = 68)	Very Low Food Security (n = 146)
Diet Quality***					
Excellent	21 (7)	7 (14)	5 (13)	4 (6)	5 (3)
Very Good	37 (12)	6 (12)	3 (8)	16 (24)	12 (8)
Good	99 (33)	22 (43)	10 (26)	23 (34)	44 (30)
Fair	96 (32)	12 (24)	18 (46)	20 (29)	46 (32)
Poor	51 (17)	4 (8)	3 (8)	5 (7)	39 (27)
Community Program Meals**	45 (15)	3 (6)	4 (10)	4 (6)	34 (23)
Places to Get Lunch^a^^b^*					
Large Grocery Store	106 (35)	19 (37)	15 (38)	27 (40)	45 (31)
Convenience Store	60 (20)	6 (12)	5 (13)	8 (12)	41 (28)
Neighborhood Market	24 (8)	8 (16)	2 (5)	6 (9)	8 (5)
Fast Food Restaurant	56 (18)	12 (24)	4 (10)	14 (21)	26 (18)
Other	58 (19)	6 (12)	13 (33)	13 (19)	26 (18)
Help with Meals^c^***					
None of the time	86 (28)	8 (16)	6 (15)	17 (25)	55 (38)
A little of the time	65 (21)	8 (16)	10 (26)	10 (15)	37 (25)
Some of the time	76 (25)	13 (25)	7 (18)	25 (37)	31 (21)
Most of the time	46 (15)	11 (22)	11 (28)	12 (18)	12 (8)
All of the time	31 (10)	11 (22)	5 (13)	4 (6)	11 (8)
Prepares most of own meals***	266 (88)	42 (82)	27 (69)	62 (91)	135 (92)
Shops for own food	272 (89)	41 (80)	34 (87)	61 (90)	136 (93)

^a^Where participants acquired foods for breakfast and dinner did not differ (p>0.05).

^b^Response “other” consists of the following responses: food bank, farmers’ market, I do not eat this meal, or other. If participants originally chose “other” for a response, we did not have a follow-up question to clarify.

^c^In response to the survey question, “How often is someone available to prepare your meals if you were unable to do this yourself?”.

P-values for chi-square tests homogeneity.

*p-value<0.05

**p-value<0.01

***p-value <0.001

Table 3 shows the results of the unadjusted and adjusted ordinal regression models. The results of the full adjusted model with all covariates included are shown in Table 1 in the Appendix. For this analysis, an odds ratio greater than one indicates an increased odds of being in the very low food security group compared with the low, marginal, and high food security groups; or of being in the very low and low food security food groups in comparison to the marginal and high security food groups; or being in the very low, low, and marginal food security groups compared to the high food security group. In the unadjusted analysis, being homeless (OR 3.16, 95% CI: 1.82, 5.48), depressed (OR 3.90, 95% CI: 2.51, 6.05), and ever using drugs (OR 1.60, 95% CI: 1.04, 2.45) were associated with a greater odds of being in a higher level food insecure group (marginal, low, or very low food security). Always having access to a car compared to never having access to a car (OR 0.33, 95% CI: 0.20, 0.56), being employed full time compared to unemployed (OR 0.45, 95% CI: 0.27, 0.76), and having help with meals all of the time compared to none of the time (OR 0.22, 95% CI: 0.10, 0.48) were each associated with a lower odds of being in a higher level of food insecurity.

**Table 3:** Multivariable odds ratios and 95% confidence intervals for food insecurity from ordinal regression models: Adults on probation in Rhode Island, 2016 (n = 304).

	Unadjusted		Adjusted	
	OR (95% CI)	p-value	AOR (95% CI)	p-value
Male	0.83 (0.52, 1.33)	0.443	------	
Race/ethnicity				
Black, non-Hispanic	1.09 (0.59, 2.01)	0.779	------	
Hispanic or Latino, any race	1.41 (0.82, 2.43)	0.213	------	
White, non-Hispanic	Referent		------	
Other	1.86 (0.91, 3.78)	0.087	------	
Homeless	3.16 (1.82, 5.48)	<0.001	2.34 (1.31, 4.18)	0.004
Car Access				
Never	Referent		------	
Sometimes	0.83 (0.50, 1.38)	0.474	------	
Always	0.33 (0.20, 0.56)	<0.001	------	
Employment Status			------	
Unemployed	Referent		------	
Part-time job	0.98 (0.57, 1.69)	0.944	------	
Full-time job	0.45 (0.27, 0.76)	0.003	------	
Depressed	3.90 (2.51, 6.05)	<0.001	3.12 (1.98, 4.91)	<0.001
Help with Meals^a^				
None of the time	Referent		Referent	
A little of the time	0.68 (0.36, 1.29)	0.241	0.77 (0.39, 1.49)	0.435
Some of the time	0.44 (0.24, 0.80)	0.007	0.54 (0.29, 0.98)	0.044
Most of the time	0.23 (0.12, 0.45)	<0.001	0.31 (0.15, 0.61)	0.001
All of the time	0.22 (0.10, 0.48)	<0.001	0.28 (0.12, 0.64)	0.003
Current drug use	1.01 (0.59, 1.72)	0.970	------	
Drug use ever	1.60 (1.04, 2.45)	0.032	------	
IDU ever	1.70 (0.95, 3.02)	0.072	------	

OR = odds ratios; CI = confidence intervals; AOR = adjusted odds ratio

^a^In response to the survey question, “How often is someone available to prepare your meals if you were unable to do this yourself?”. Response options were: “none of the time”, “a little of the time”, “some of the time”, or “most of the time”.

IDU = Injection Drug Use

The second column of Table 3 shows the final adjusted ordinal regression model. Being homeless (AOR 2.34, 95% CI: 1.31, 4.18) and depressed (AOR 3.12, 95% CI: 1.98, 4.91) were independently associated with a greater odds of being in a higher level food insecure group. Compared to having help with meals none of the time, participants that had meal help all of the time (AOR 0.28, 95% CI: 0.12, 0.64), most of the time (AOR 0.31, 95% CI: 0.15, 0.61), and some of the time (AOR 0.54, 95% CI: 0.29, 0.98) had a lower odds of being in a higher level food insecure group. Always having access to a car and a full-time job were associated with a lower odds of being in a more food insecure level in the unadjusted models, but this relationship was attenuated and not statistically significant in the adjusted model, and therefore removed from the final model for parsimony.

**Fig 3:**
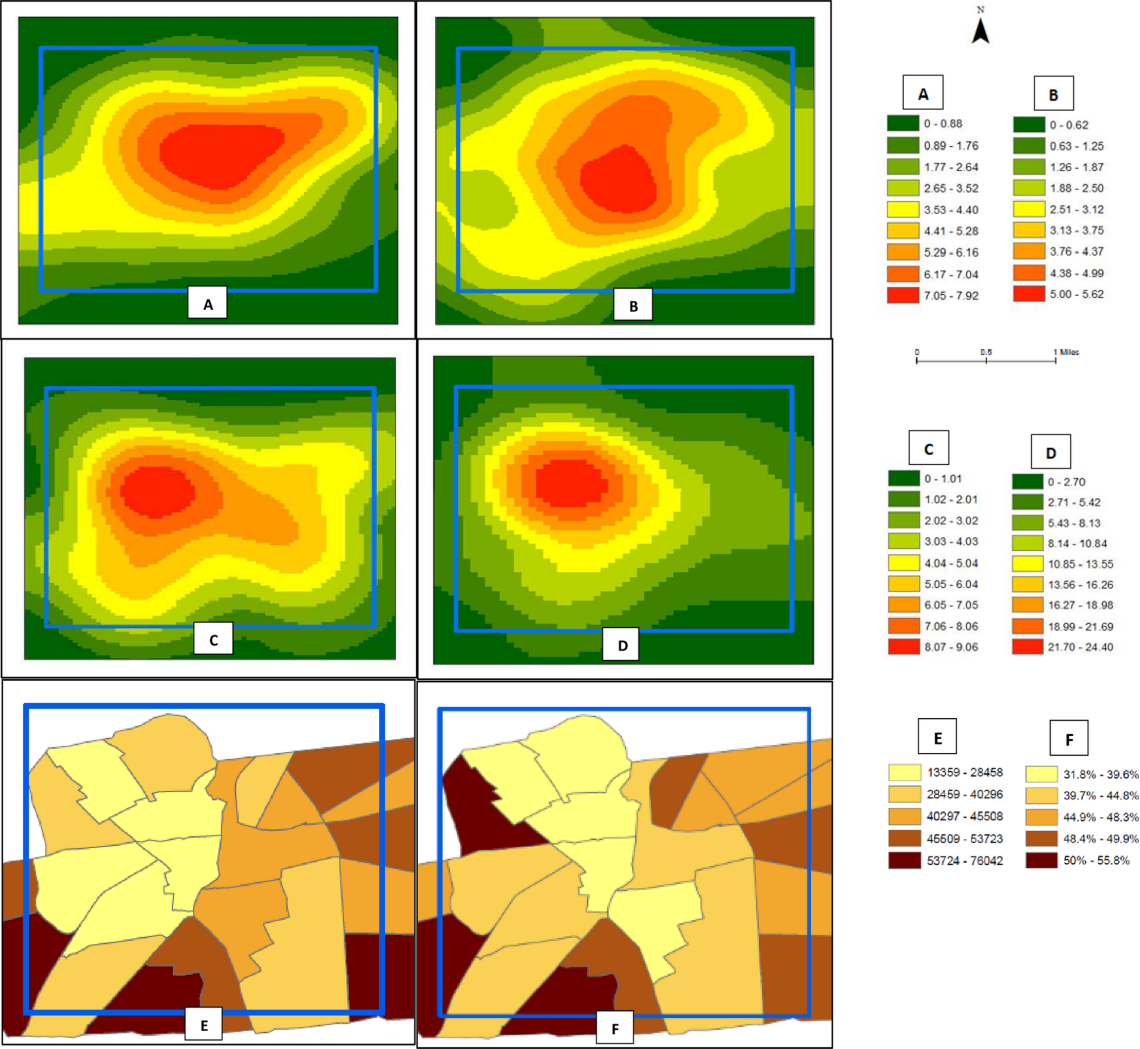
Kernel density estimates of probationers place of residence by food security status, 2016. **(**A) Presents where participants with high food security reside. (B) Presents where participants with marginal food security reside. (C) Presents where participants with low food security reside. (D) Presents where participants with very low food security reside. The counts for each ramp represent the number of people per square mile. (E) Presents the median income ($) of the general population by quintiles at the census tract level. (F) Presents the employment rate of adults aged 16–64 years of the general population by quintiles at the census tract level. The blue box provides a study frame to reference the Kernel density estimates. This study frame was selected to include the majority of participants that lived within the catchment area for the specific probation office that was the venue for the study.

We geocoded the residence of the 267 participants (88%) who provided an accurate address or intersection. Only one participant refused to provide information about place of residence and one participant did not know his/her address. The other “missing” addresses or intersections were incorrectly provided. Heat maps that present the result of Kernel density estimates based upon participants’ place of residence by level of food security status are presented in Fig 3. The geospatial distribution (i.e., the density) of study participants varied by food security status. A higher density of very low and low food security participants resided in the northwest region of the study catchment area in contrast to marginal and high food security participants, who lived in higher densities in the central region of the study catchment area. The census tracts with the lowest median incomes of the general population (light yellow in panel E) are in the western and northwestern parts of the study catchment area, which is the same area with the highest density of very low and low food security from our study population (red in panels C and D). The highest density of all levels of food security (red in panels A, B, C, and D) are within the census tracts with the lowest rates of full-time employment in the general population (lightest yellow in panel F).

## Discussion

Our study is among the first to empirically assess food insecurity and food acquisition methods in adults on probation. We found that more than two-thirds of our study population were food insecure, suggesting that access to quality foods on a regular basis is a common problem for individuals on probation. The prevalence of food insecurity and very low food security in our study participants were both substantially higher compared to the general population within the state of Rhode Island (with different periods of assessment, 30 days versus 12 months, respectively). There are no data available for each of the ten food insecurity indicators for the general population of Rhode Island (as in Fig 2); however, this is reported nationally in the 2016 USDA Household Food Security report.[1] According to this report, approximately one-third (33%) of US households with very low food security reported an adult not eating for an entire day and 26% reported that this occurred in three or more months during the year.[1] Almost half (44%) of US households with very low food security reported weight loss because there was not enough money for food.[1] These percentages are high nationally, but even higher in our study population. The USDA Household Food Security report provides this information over a 12-month reporting period. Given the unstable nature of our probation population and to assess current food security status, we used a 30-day reporting period. Food insecurity fluctuates throughout the year and prior studies have shown that the prevalence of food insecurity in the past 30 days is often lower compared to prevalence over the prior year[1, 37] or that the prevalence of food insecurity increases as the length of the observation period increases,[38] which makes the findings in our study population even more striking.

Our findings of high rates of food insecurity are consistent with other studies investigating the prevalence of food insecurity among other vulnerable populations, such as those who are homeless and substance users.[19, 20, 39–42] In homeless populations in resource-rich countries like the US, the reported prevalence of food insecurity ranged from 49–94%.[19, 20] For people who inject drugs, the prevalence of food insecurity has been reported in the range of 58–71%;[41, 43] for illicit non-injection drug use, the prevalence is 34–36%.[42, 44]

The probationers in the current study have profiles that put them at risk for poorer health such as high rates of smoking and histories of substance and alcohol use. According to the 2012 National Survey on Drug Use and Health, the prevalence of alcohol or illicit drug use disorder in the past year was 40.3% in male probationers.[45] About one-fourth of participants in the current study have problems with hazardous drinking. We hypothesize that the 19% prevalence of reported current drug use may be an underestimate given the study venue; although probation officers were not present during the questionnaire administration, participants may have been fearful of reporting illicit drug use at a probation office.

The National Commission on Hunger, a committee established by Congress, released a report in January 2016 to provide recommendations to improve the programming of existing programs and USDA funding to address hunger and food insecurity.[46] Among the specific populations that experience high rates of hunger identified by the Commission were people affected by high incarceration rates.[46] This report highlighted the need to address the root causes of hunger in America, such as underemployment, unstable families, insufficient education, exposure to violence, a history of racial or ethnic discrimination, personal choices, or a combination of these factors. Our study findings underscore the importance of the Commission’s recommendations, particularly addressing the root causes of hunger (food insecurity), since many in our probation population experience the root causes of food insecurity identified by the Commission. Food insecurity is often compounded by other issues of financial hardship, especially with high rates of unemployment and underemployment, faced by this population to be able to pay for safe housing, healthcare, and other resources that contribute to wellbeing.

Although three quarters of study participants received SNAP, these benefits were not sufficient to address the high prevalence of food insecurity in our population. This marginalized population has limited resources and choices for food across all levels of food security. Economic constraints may make food less of a priority compared to securing housing, drugs, alcohol, cigarettes, or other resources. Participants acquired food primarily from large grocery stores, convenience stores, and fast food restaurants. Although we did not assess their dietary quality, we did capture the participants’ perceptions of their dietary quality. About half of our participants rated the quality of their diet to be fair or poor and the majority of participants that perceived their diet quality to be poor had very low food security. Our current food system needs structural changes to temper the low access, high cost, and poorer dietary quality experienced by this population. Understanding meal patterns and food acquisition methods can inform practical guidance to provide information on how to provide healthy, accessible, and low cost foods to this population.

In addition to improved food access, it remains important to help this population make the connection between the urgency to improve dietary quality with limited resources and prevention of the onset or exacerbation of chronic disease. About one-quarter of our study participants were homeless. Community food sources, like food banks, provide public social support but may not be an ideal food source for homeless individuals because they may not have access to a kitchen to prepare and properly store foods. Other community food resources that provide public social support, such as congregate meal sites, provide a good option for addressing food sustenance, although dietary quality may be variable, and availability is often limited to one meal per weekday, which leaves a gap in the number of meals individuals can procure. Public meal support that is acceptable and realistic for this population in light of their limited resources should be explored. Individuals involved with the correctional system may experience low levels of support due to stigma or because they avoid social groups associated with former criminogenic environments.

The relationship between food insecurity and depression has been explored in several studies. In studies that used the USDA Household Food Security Module and CES-D to assess depression (similar to our study), food insecurity has been found to be an independent correlate of depression[42, 47, 48] and depression has been found to be an independent correlate of food insecurity.[49–53] These studies highlight the complicated bidirectional relationship between food insecurity and depression and future studies are warranted to determine the efficacy of intervening on food insecurity or depression to alleviate the other. In general, our study participants resided in areas with the lowest rates of full-time employment in the general population. Higher densities of food insecure participants resided in census tracts with the lowest median income in the general population. This further indicates the poverty of our study population and that material hardships may make prioritizing and accessing food a challenge. Future research that looks more closely at geospatial associations between food insecurity, probation status, and other covariates is merited.

To our knowledge, ours is the first study to quantify food insecurity and identify food acquisition methods in probationers. The variety of potential correlates assessed allowed for a thorough investigation of food insecurity among probationers. We identified some limitations of this study. First is the inability to assess temporal relationships due to its cross-sectional study design. Second, we have potential limited generalizability because we focused on a convenience sample in one probation office in a small state. However, our participants were representative of the sociodemographics of this probation office population, based upon the estimation of the probation officers, and Rhode Island has the second highest rate of probationers in the country. Third, there may have been issues with social desirability bias given the nature of the sensitive questions we asked and the location of our study at the probation office. We used the ACASI for survey administration in hopes of eliciting more honest responses and probation officers reminded participants that information collected would be confidential.

## Conclusion

The prevalence of food insecurity and very low food security among adults on probation was extremely high compared to the general population, indicating a critical need for action. We identified characteristics of probationers most at risk for food insecurity, which includes being homeless and depressed and lacking social support for meal preparation. These findings can inform development of targeted interventions and policies to mitigate health disparities in this population. The strategies to improve food insecurity need to confront the root causes of social determinants, such as improving affordable access to healthier foods, and structural determinants, such as addressing barriers to gainful employment, safe housing, and treatment for depression, in order to reduce the criminogenic risk factors for people on probation. There are many individuals on probation in the US, so this provides a window of opportunity to intervene to improve the wellbeing of a vulnerable population.

## Supporting information

S1 Table Multivariable odds ratios and 95% confidence intervals for food insecurity from ordinal regression models, full model: Adults on probation in Rhode Island, 2016 (n = 304). (DOCX)
