# Supplementary material for: Food acquisition methods and correlates of food insecurity in adults on probation in Rhode Island
Source: PLoS One. 2018 Jun 8;13(6):e0198598. doi: 10.1371/journal.pone.0198598 (PMC5993252; doi:10.1371/journal.pone.0198598)
Supplement: S1 Table — (DOCX) [file pone.0198598.s001.docx]

|  | **Unadjusted** | | **Adjusted** | |
| --- | --- | --- | --- | --- |
|  | OR (95% CI) | p-value | AOR (95% CI) | p-value |
| Male | 0.83 (0.52, 1.33) | 0.443 | 0.91 (0.54, 1.54) | 0.729 |
| Race/ethnicity |  |  |  |  |
| Black, non-Hispanic | 1.09 (0.59, 2.01) | 0.779 | 0.99 (0.49, 2.00) | 0.981 |
| Hispanic or Latino, any race | 1.41 (0.82, 2.43) | 0.213 | 1.43 (0.78, 2.64) | 0.244 |
| White, non-Hispanic | Referent |  | Referent |  |
| Other | 1.86 (0.91, 3.78) | 0.087 | 1.32 (0.59, 2.94) | 0.500 |
| Homeless | 3.16 (1.82, 5.48) | <0.001 | 1.97 (1.04, 3.72) | 0.038 |
| Car Access |  |  |  |  |
| Never | Referent |  | Referent |  |
| Sometimes | 0.83 (0.50, 1.38) | 0.474 | 1.24 (0.71, 2.15) | 0.453 |
| Always | 0.33 (0.20, 0.56) | <0.001 | 0.69 (0.37, 1.29) | 0.249 |
| Employment Status |  |  |  |  |
| Unemployed | Referent |  | Referent |  |
| Part-time job | 0.98 (0.57, 1.69) | 0.944 | 1.18 (0.64, 2.19) | 0.595 |
| Full-time job | 0.45 (0.27, 0.76) | 0.003 | 0.94 (0.51, 1.72) | 0.829 |
| Depressed | 3.90 (2.51, 6.05) | <0.001 | 3.13 (1.94, 5.05) | <0.001 |
| Help with Meals^a^ |  |  |  |  |
| None of the time | Referent |  | Referent |  |
| A little of the time | 0.68 (0.36, 1.29) | 0.241 | 0.77 (0.38, 1.53) | 0.452 |
| Some of the time | 0.44 (0.24, 0.80) | 0.007 | 0.54 (0.29, 1.04) | 0.064 |
| Most of the time | 0.23 (0.12, 0.45) | <0.001 | 0.31 (0.15, 0.64) | 0.001 |
| All of the time | 0.22 (0.10, 0.48) | <0.001 | 0.29 (0.12, 0.68) | 0.005 |
| Current drug use | 1.01 (0.59, 1.72) | 0.970 | 0.64 (0.32, 1.25) | 0.190 |
| Drug use ever | 1.60 (1.04, 2.45) | 0.032 | 1.31 (0.77, 2.23) | 0.314 |
| IDU ever | 1.70 (0.95, 3.02) | 0.072 | 1.34 (0.66, 2.72) | 0.413 |
